# Durvalumab-Induced Demyelinating Lesions in a Patient With Extensive-Stage Small-Cell Lung Cancer: A Case Report

**DOI:** 10.3389/fphar.2021.799728

**Published:** 2022-01-03

**Authors:** Wenhui Liu, Bo Chen, Yiping Liu, Zhiying Luo, Bao Sun, Fang Ma

**Affiliations:** ^1^ Department of Pharmacy, The Second Xiangya Hospital, Central South University, Changsha, China; ^2^ Institute of Clinical Pharmacy, Central South University, Changsha, China; ^3^ Department of Oncology, The Second Xiangya Hospital, Central South University, Changsha, China; ^4^ Department of Pharmacy, The Central Hospital of Yongzhou, Yongzhou, China

**Keywords:** small-cell lung cancer, durvalumab, neurological demyelination lesions, immune-related adverse events, immune checkpoint inhibitors

## Abstract

It is of great clinical value to investigate the immune-related adverse events (irAEs), especially demyelinating lesions, caused by immune checkpoint inhibitors (ICIs). The incidence of demyelinating lesions is less frequent in irAEs, but once it occurs, it will seriously affect the survival of patients. The present study reports a case of durvalumab-induced demyelinating lesions in a patient with extensive-stage small-cell lung cancer. Subsequently, the patient receives a high intravenous dose of methylprednisolone and his condition is improved after 21 days of treatment. Altogether, early diagnosis and treatment of ICIs-related neurological irAEs is of great significance to the outcome of the patient’s condition.

## Introduction

With the advent of ICIs, the occurrence and prevention of irAEs has drawn more and more attention. ICIs exert anti-tumor effects by activating T cells and enhancing the systemic immune response, while they may also cause systemic or local inflammation and autoimmunity, as well as direct or indirect neurotoxicity (Gu et al., 2017). The total incidence of neurological irAEs was 3.8–12%, and the cumulative incidence of severe neurotoxic reactions was less than 1% in patients receiving ICIs monotherapy or combination therapy, which contributed to severe or even fatal consequences (Hodi et al., 2010; Larkin et al., 2015; Cuzzubbo et al., 2017; Choi and Lee, 2020; Duong et al., 2021). ICIs-induced demyelinating lesions, one of the neurological irAEs, mainly manifested multifocal and inflammatory demyelination (van den Berg et al., 2014). The main characteristics of the pathological process were demyelination involved in brain, spinal cord and peripheral nerves, and myelin damage or loss (Goodfellow and Willison, 2016; Donofrio, 2017). More importantly, the clinical manifestations and pathogenesis of the neurological irAEs were complex, and their response to treatment greatly varied among individuals, which posed great challenges to clinical treatment.

## Case Presentation

**FIGURE 1:**
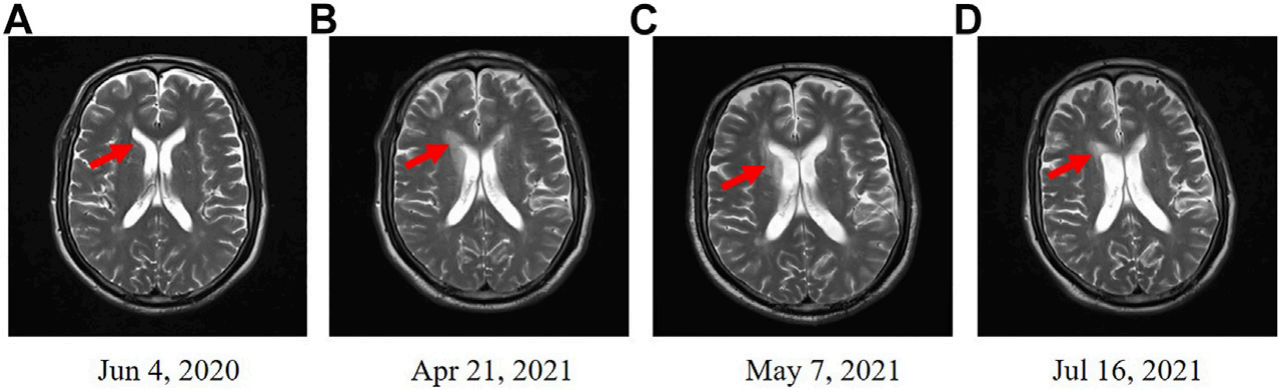
Enhanced MRI scans in head. **(A)** Enhanced MRI before the treatment of durvalumab. **(B)** Enhanced MRI revealed the diffuse abnormal signal after a 11-cycle monotherapy maintenance therapy with durvalumab. **(C)** Enhanced MRI revealed an improved condition after 21 days treatment of methylprednisolone. **(D)** Enhanced MRI indicated that the diffuse abnormal signal was disappeared after 3 months.

**FIGURE 2:**
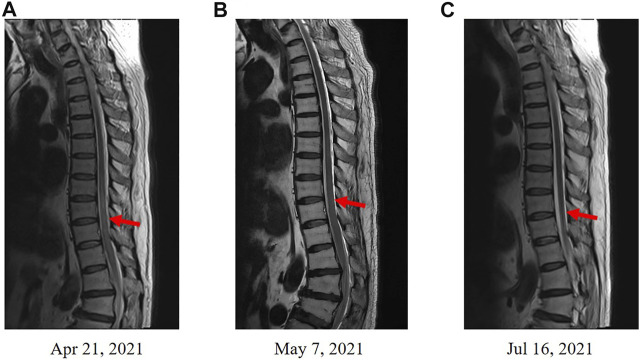
Enhanced MRI scans in spinal cord. **(A)** Enhanced MRI before the treatment of methylprednisolone. **(B)** Enhanced MRI revealed an improved condition after the treatment of methylprednisolone. **(C)** Enhanced MRI revealed a disappeared diffuse abnormal signal after 3 months.

In June 2020, a 67-year-old male with a history of smoking (smoking index at 1,600) was diagnosed with extensive-stage small-cell lung cancer (TNM stage IV), along with pleural metastasis. The patient was initially treated with six cycles of EP (Etoposide + Nedaplatin) program (Etoposide 170mg, d1-3, Nedaplatin 140mg, d1) combined with durvalumab (500 mg) chemotherapy (Figure 1A), and chest computed tomography (CT) scan confirmed a partial response. Subsequently, the patient received a 11-cycle monotherapy maintenance therapy with durvalumab. However, after a 10-cycle monotherapy maintenance therapy, he experienced increased numbness of limbs, weakness of both lower limbs, and dysuria. Chest CT scan observed a stable disease, with a PFS of 10.1 months. After receiving mecobalamin and vitamin B1, the symptoms did not improve significantly. Enhanced magnetic resonance imaging (MRI) found the diffuse abnormal signal in both head and spinal cord (Figures 1B, 2A). Biochemical analysis indicated that the level of glucose and chloride was normal, while the protein was elevated in cerebrospinal fluid (CSF) (Table 1). Furthermore, the presence of CSF-specific oligoclonal bands was positive. Next generation sequencing (NGS) did not find viruses and pathogens in the CSF. In addition, electromyography showed that the patient’s limbs had peripheral neurogenic damage and electrophysiological changes, as well as multiple injuries in the motor and sensory nerves. According to 2021 National Comprehensive Cancer Network (NCCN) Guidelines [(2021).Guideli, 2021] and the clinical diagnostic criteria of acute inflammatory demyelinating polyneuropathies (AIDP) and multi-disciplinary treatment (MDT) (Alessandro et al., 2018; Guidon et al., 2021), the final diagnosis of the patient was demyelinating lesions associated with durvalumab, namely Guillain-Barre syndrome (GBS).

**TABLE 1:** Biochemical analysis of glucose, chloride and protein in cerebrospinal fluid.

CSF index	Values^a^	Reference values
Protein (mg/L)	685	150–450
Glucose (mmol/L)	3.05	2.5–4.5
Chloride (mmol/L)	127.3	120–132

CSF, cerebrospinal fluid.

a Values were tested at April 21, 2021.

**FIGURE 3:**
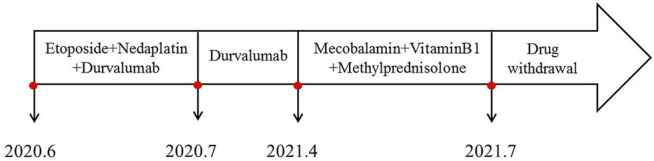
Timeline of treatment administration from the episode of care.

In May 2021, the patient received methylprednisolone (1,000 mg, d1) for the durvalumab-induced demyelinating lesions. After 21 days, the demyelinating lesions were improved than before (Figures 1C, 2B), and the dose of methylprednisolone was gradually reduced until it was stopped. Three months later, Enhanced MRI indicated that the diffuse abnormal signal was disappeared (Figures 1D, 2C), and the symptoms of numbness and fatigue of the patient’s limbs were completely disappeared. Until the last follow-up in August 2021, the patient had not shown any symptoms of numbness and fatigue, as well as obtained a durable response. The timeline treatment administration from the episode of care was presented in Figure 3.

## Discussion

In this study, we presented a successful case of a patient with small cell lung cancer who received durvalumab therapy. The patient showed the initial symptoms of numbness and fatigue after the treatment of durvalumab monotherapy. Further enhanced MRI, biochemical analysis and NGS of CSF, as well as electromyography excluded the possibility of tumor-induced encephalopathy such as paraneoplastic neurological syndrome, metastatic blain tumors, and cancerous meningitis and confirmed that the diagnosis of the patient was GBS. Subsequently, the patient received high-dose shock therapy with methylprednisolone (1,000 mg, d1), and the symptoms were completely improved.

Durvalumab is an ICI, and its related adverse reactions involve organs or systems such as the skin, digestive tract, liver, lung, thyroid, and pituitary. To the best of our knowledge, this is the first case that showed the GBS caused by durvalumab. Of note, GBS, one of a serious type of neurotoxic irAEs, has a low incidence and high mortality. Supakornnumporn et al. (Supakornnumporn and Katirji, 2017) reported six patients with GBS after receiving ICIs treatment. Among them, three cases died, two cases improved, and one case maintained symptom. To date, many literatures have reported the treatment of neurotoxic irAEs with glucocorticoids and even intravenous immunoglobulin (IVIG) or plasmapheresis (Jacob et al., 2016; Donofrio, 2017; Schneiderbauer et al., 2017; Supakornnumporn and Katirji, 2017). According to the irAEs classification in the 2021 National Comprehensive Cancer Network (NCCN) treatment Guidelines ((2021).Guideli, 2021), this patient was grade 3 and should be treated with high-dose glucocorticoid shock therapy.

GBS is the most severe acute paralytic neuropathy and is usually preceded by infection or immune stimulation. However, the pathogenesis of GBS remains elusive. A previous study reported that molecular mimicry and antiganglioside antibodies are involved in the pathogenesis of GBS(van den Berg et al., 2014). On the other hand, Willison et al. suggested that GBS was mainly a humorally-mediated, rather than T-cell-mediated disorder, at least in the progressive phase of nerve injury (Willison et al., 2016). Further studies are needed to figure out the exact mechanism of GBS.

Nevertheless, there exist some limitations in our report. Studies have shown that neurological irAEs occur more frequently in the induction phase of patients with ICIs treatment (Zimmer et al., 2016), which is consistent with our case. However, there is no unified standard for the detection of ICIs-induced demyelinating lesions at the present, and the diagnosis mainly relies on enhanced MRI, electromyography and other biochemical analysis, which suggests that further researches or guidelines that standardize the unified standard for ICIs-induced detection are needed in the future.

Finally, high-dose glucocorticoid shock therapy should be the first choice for the treatment of ICIs-related demyelinating lesions, but it is also necessary to alert the further deterioration of symptoms at the initial stage of treatment. Early diagnosis and treatment is of great significance to the outcome of the patient’s condition. Plasmapheresis or IVIG may be preferred if the patient’s symptoms do not improve or if the patient develops severe neurotoxic irAEs.

## Data Availability

The original contributions presented in the study are included in the article/supplementary material, further inquiries can be directed to the corresponding authors.
